# Supplementary figures and images for: Efficacy of Light-Emitting Diode-Mediated Photobiomodulation in Tendon Healing in a Murine Model
Source: Int J Mol Sci. 2025 Mar 4;26(5):2286. doi: 10.3390/ijms26052286 (PMC11899806; doi:10.3390/ijms26052286)

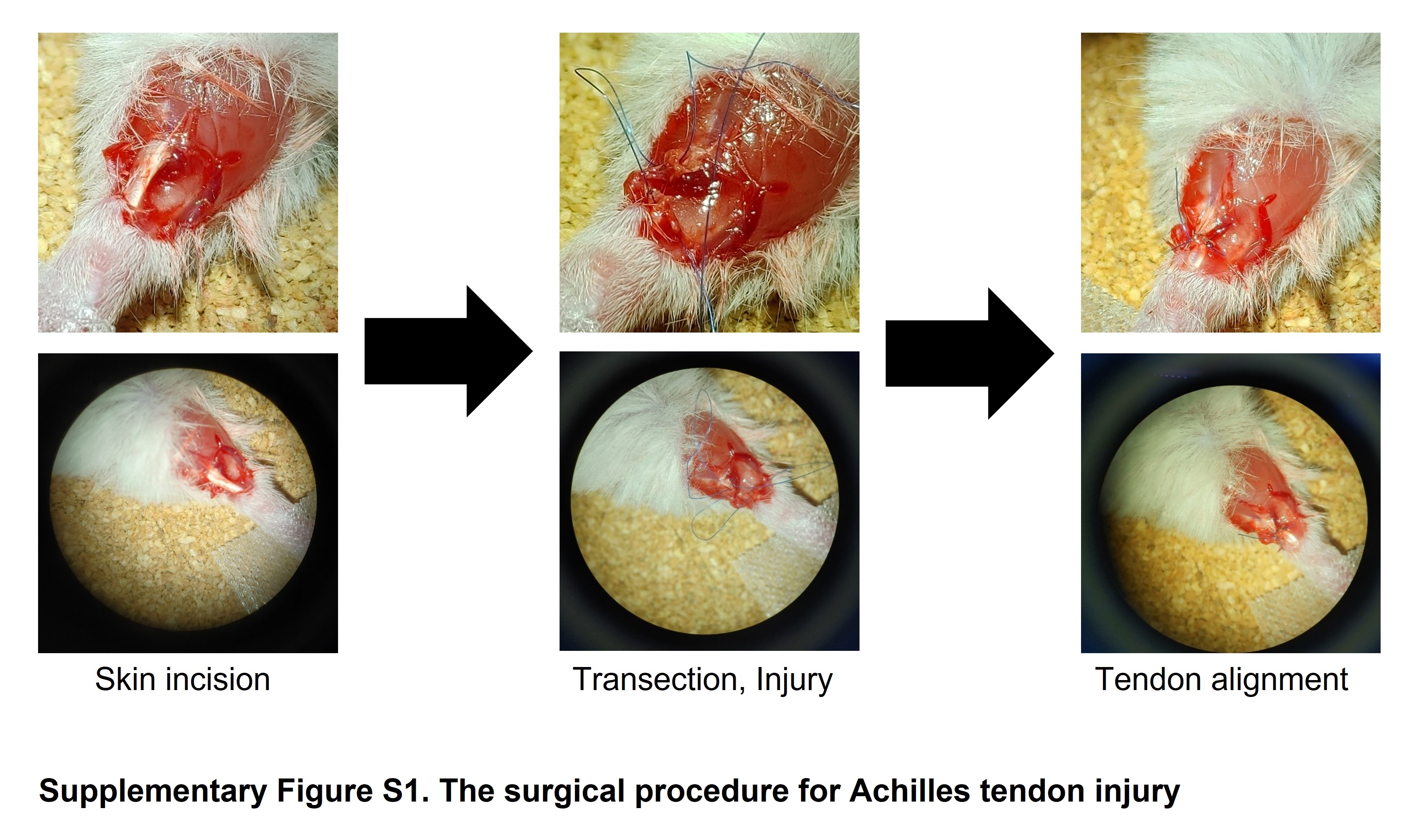

Supplement: Supplementary file 1 [file ijms-26-02286-s001.zip › ijms-3482234-supplementary.jpg]
